# Less micrometastatic risk related to circulating tumor cells after endoscopic breast cancer surgery compared to open surgery

**DOI:** 10.1186/s12885-019-6158-3

**Published:** 2019-11-08

**Authors:** Shichao Li, Wenting Yan, Xinhua Yang, Li Chen, Linjun Fan, Haoxi Liu, Kun Liu, Yi Zhang, Jun Jiang

**Affiliations:** 1 Department of Breast and Thyroid Surgery, Southwest Hospital, Army Medical University (Third Military Medical University), Gaotanyan Street 29, Chongqing, 400038 China; 2 Breast and Thyroid Disease Center, Third Affiliated Hospital of Chongqing Medical University, Triumphant Road, Yubei District, Chongqing, 401120 China; 3 Department of Epidemiology, College of Preventive Medicine, Army Medical University (Third Military Medical University), Chongqing, 400038 China

**Keywords:** Breast neoplasms, Neoplastic cells, Circulating tumor cells, Micrometastases, Endoscopic breast surgery, Open surgery

## Abstract

**Background:**

Increase of circulating tumor cells (CTCs) has been found after surgery for various carcinomas but not confirmed for breast cancer, and whether endoscopic surgery confers identical effect to CTCs as open surgery did is not clear. The present study aimed to investigate whether CTCs increase after surgery and whether there is a difference between open surgery and endoscopic surgery.

**Methods:**

Pre- and postoperative peripheral blood (5 mL) obtained from 110 female patients with operable breast cancer (53 underwent endoscopic surgery, 57 underwent open radical mastectomy). Quantitative real-time reverse transcription-PCR was done to detect cytokeratin 19 mRNA-positive CTC. CTC detection rate, cell number and the increase after surgery (named micrometastasis) were compared between the two groups.

**Results:**

In the open group, CTC positive rate before and after surgery were 22.81 and 33.33%; median CTC number before and after surgery were 0.21 and 0.43 and 17 patients (29.82%) had increased micrometastatic risk. In the endoscopic group, CTC positive rate before and after surgery were 24.53 and 28.30%; median CTC number before and after surgery were 0.27 and 0.36, and 8 patients (15.09%) had increased micrometastatic risk. There was a suggestive higher postoperative CTC detection rate and CTC number and a significant increased postoperation micrometastatic risk was observed in the open group compared to the endoscopic group (OR = 3.19, 95%CI: 1.05–9.65) after adjustment for clinicopathologic characteristics.

**Discussion:**

CTC tends to increase in breast cancer patients after surgery, and the micrometastatic risk was higher for open surgery compared to endoscopic surgery.

**Trial registration:**

This study was prospectively registered at Chinese Clinical Trial Register (ChiCTR-OCH-10000859, 24 April 2010).

## Background

Breast cancer is by far the most common malignancy in women and it is ranked as the leading cause of cancer death [1]. However, relapse and metastasis are still the primary reason for mortality, which mainly result from micrometastasis undetected by conventional approaches in blood, bone marrow or lymph nodes. Recent clinical researches have focused on detection and exploration of micrometastasis in the form of circulating tumor cells (CTC) in peripheral blood, which significantly contributes to distant metastasis [2]. Results from studies have showed a clinical value of detecting and monitoring levels of CTC for evaluation of prognosis and therapeutic regime, as well as providing reference information for clinical decision-making [3–5]. Evidence from previous clinical investigations showed that tumor surgical manipulations induce tumor cell dissemination during operation of breast, hepatic, colorectal, cervical and prostatic cancers [6–12], even leading to an increased incidence of distant metastases according to further results of animal studies [13]. In contrast to traditional open surgery, although endoscopic breast cancer surgery is capable of achieving local disease control in the majority of patients, it is considered that special procedures in endoscopic performance, including lipolysis solution, liposuction and endoscopic axillary dissection and so on, might increase the possibility of hematogenous dissemination of tumor cells, and there is a lack of evidence in support of the oncological security of this technique [14]. Accordingly, the aim of this pilot study was to evaluate the effect of the surgical approach on micrometastatic risk correlated to surgery.

Detection of CTC before and after resection of primary breast tumor might be helpful to expand our knowledge about the effect of surgery on dissemination of malignant cells. One of the most frequently used markers for the detection of CTCs in the PB of patients with breast cancer is cytokeratin-19 (CK-19), a cytoskeletal protein expressed on epithelial but not on mesenchymal cells, that is expressed on virtually all breast cancer cells. Previous studies have reported that the detection of CTCs by real-time reverse transcription-polymerase chain reaction (RT-PCR) based on detection of CK-19 mRNA provides a sensitive, accurate approach and allows for reliable quantification of mRNA transcripts in an individual sample [15, 16].

## Methods

### Patients

Between 2010 and 2011, a total of 110 female patients with newly diagnosed breast cancer (stageI-III) treated with primary surgery at the Breast Disease Center of Southwest Hospital were prospectively enrolled. A preoperative diagnosis of breast carcinoma by core needle biopsy was required. Before surgery, a complete baseline diagnostic evaluation for the detection of distant metastases was performed, including chest X-rays, liver function tests, ultrasound of the liver and whole-body bone scan when indicated. Patients with distant metastases or open biopsy prior to operation were excluded. The choice of open or endoscopic approach depended on the surgeon to whom the patient was referred, and surgery was performed or supervised by an experienced surgeon. Fifty three of these 110 patients underwent endoscopic subcutaneous mastectomy and axillary dissection, including 20 patients who received immediate breast reconstruction with implant or latissimus dorsi flap and 33 patients who just received endoscopic nipple and/or skin sparing subcutaneous mastectomy first and will received breast reconstruction at a second stage. And 57 patients received open radical mastectomy. There was no difference in the protocol of anesthesia between the two groups. In endoscopic procedures, axillary dissection was performed before subcutaneous mastectomy and an endoscopic work space was maintained by inflation of the subcutaneous tissue using carbon dioxide at a pressure of 8-10 mmHg (1 mmHg = 1.33 kPa). The axillary lymph node dissection was also done for the open surgery cases, although mastectomy is performed followed by axillary dissection in the routine open modified radical mastectomy, while in the endoscopic procedure, axillary dissection is prior to mastectomy. Among the 53 microscopic surgery cases, 20(37.7%) received immediate breast reconstruction.

All the patients also participated in a prospective study for early diagnosis of recurrence, metastasis and prognosis monitoring by circulating breast-tumor cells detecting (ChiCTR-OCH-10000859). Tumor stage was classified according to the sixth edition of the TNM classification of the American Joint Commission on Cancer (6th edition). Written informed consent for the collection of blood samples was obtained from all participating patients and the protocol of this study was approved by the Ethical Committee of the Southwest Hospital (KY201003).

### Blood samples, real-time RT-PCR assay for CK-19 mRNA detection

Five mL peripheral blood was drawn from every patient prior to surgery (t0) and 12 h after the operation (t1) and all laboratory analyses were conducted in a blinded fashion. Mononuclear cells, including tumor cells, were harvested by gradient density centrifugation using Ficoll-Hypaque. Total RNA isolation was carried out by using TRNzol reagent (Tiangen Biotech, Beijing, China) according to the manufacturer’s instructions. All RNA preparations and handling steps took place in a laminar flow hood under RNAse-free conditions. The isolated RNA was dissolved in diethylpyrocarbonate-treated water and stored at − 80 °C for future use. The purity and quantity of RNA were determined using a spectrophotometer (260 nm/280 nm). Reverse transcription of RNA was carried out with the PrimeScript™ RT reagent (TOYOBO, Japan). Two μg of total RNA was used as template to synthesize cDNA according to the manufacturer’s instructions. The real-time RT-PCR assay for CK-19 mRNA-positive CTCs has been previously described in detail elsewhere (SYBR Green Realtime PCR Master Mix, TOYOBO, Japan). The number of CTCs in each of the tested samples was expressed as Michigan Cancer Foundation-7 (MCF-7) breast cancer cell line equivalents per 2 μg of total-RNA, as determined by LightCycler system (Roche Diagnostics, Germany) software 3.1, according to the external standard calibration curve. According to the analytic detection limit of our assay, the presence of ≥0.9 MCF-7 cell equivalents/2 μg of total RNA was considered a positive result of CTCs, following the protocol of Stathopoulou et al. [17] Using this detecting limit as a cut-off, surgery is considered to implement an increased micrometastatic risk if the number of CTCs is increased beyond this cut-off after operation. Moreover, all blood samples were simultaneously tested for the integrity of RNA by demonstrating positive detection of the house keeping β-actin gene.

### Statistical analysis

The Mann-Whitney test was used to compare the non-normally distributed number of CTCs between different surgical groups. The Wilcoxon test was used for paired comparison of the CTC numbers before and after surgery. Unpaired comparison of binomial variables was done using the Chi-square test or Fisher exact test (when the prerequisite of Chi-square test was not fulfilled). The paired comparison of binomial variables was done using the McNemar test. Unpaired comparison of ordinal variables between the two surgical groups was done using the Mann-Whitney test. Mantel-Haenszel stratified analysis was done to exclude the potential confounder bias introduced by tumor size, lymph node status or tumor stage. Propensity score matching was done with control for age, menopausal status, tumor size, lymph node, tumor stage, histology type, estrogen receptors status, progesterone receptors status, HER-2 status and pre-operation CTC status. The matching error was set to 0.1. Then Mc Nemar test was done for comparing the micrometastatic status between the matched two groups. Multi-variate analysis for the association of surgical type and micrometastatic risk was done by binary logistic regression (backward likelihood method), with adjustment for age (transformed into ordinal variable by optimal scaling analysis), menopausal status (premenopausal V.S. postmenopausal), tumor size (T_1_ to T_3_), lymph node (pN_0_ to pN_3_), tumor stage (I to III), histology type (Invasive ductal V.S. other type), estrogen receptors status (− V.S. +), progesterone receptors status (− V.S. +), HER-2 status (− to +++) and pre-operation CTC status (negative V.S. positive). The entry and removal probability for stepwise was set as default value (0.05 and 0.10, respectively). Furthermore, the interaction of surgical type and pre-operation CTC status was additionally analyzed by binary logistic regression, too. Data analysis was carried out with PASW Statistical software version 18.0 (SPSS Inc. Chicago, IL) and Review Manager 5.0 (The Cochrane Collaboration, Copenhagen).

## Results

### Patient characteristics

A total of 110 female patients with breast cancer were recruited. The median age at diagnosis was 46 years, ranged from 32 to 60 years. Fifty-seven patients were in the open group, whereas 53 were in the endoscopic group. The characteristics were not different between the two groups (Table 1). Compared to the microscopic surgery, the open surgery had a shorter surgery duration (*P* < 0.001) but similar bleeding volume (*P* = 0.766). Nevertheless, neither surgery duration nor bleeding volume showed statistically significant difference between the micrometastatic patients and the non-micrometastatic patients (*P* > 0.05, respectively; see Additional file 1: Table S1).

**Table 1:** Characteristics of the recruited patients

Characteristic	Endoscopic group (*n* = 53)	Open group (*n* = 57)	*P* value
Age	45(33–60)	48(32–60)	0.245
Menopausal status			0.391
Premenopausal	34(64.15%)	32(56.14%)	
Postmenopausal	19(35.85%)	25(43.86%)	
Tumor size			0.100
T_1_	44(83.02%)	40(70.18%)	
T_2_	9(16.98%)	15(26.32%)	
T_3_	0(0%)	2(3.51%)	
Lymph node (pN)			0.108
pN_0_	33(62.26%)	28(49.12%)	
pN_1_	15(28.30%)	18(31.58%)	
pN_2_	4(7.55%)	7(12.28%)	
pN_3_	1(1.89%)	4(7.02%)	
Tumor stage			0.180
I	29(54.72%)	26(45.61%)	
II	19(35.85%)	19(33.33%)	
III	5(9.43%)	12(21.05%)	
Histology type			0.194
Invasive ductal	49(92.45%)	56(98.25%)	
Other	4(7.55%)	1(1.75%)	
Estrogen receptors status			0.473
ER(+)	32(60.38%)	30(53.57%)	
ER(−)	21(39.62%)	26(46.43%)	
Progesterone receptors status			0.776
PR(+)	26(49.06%)	29(51.79%)	
PR(−)	27(50.94%)	27(48.21%)	
HER-2 status			0.216
HER-2(−)	27(50.94%)	36(64.29%)	
HER-2(+)	8(15.09%)	7(12.50%)	
HER-2(++)	11(20.75%)	5(8.93%)	
HER-2(+++)	7(13.21%)	8(14.29%)	
Surgery duration, min	220(146–479)	125 (66–384)	<0.001
Bleeding volume, mL	100(30–800)	100(30–400)	0.766

Represented as “median (range)” or “frequency (percentage)”

Comparison between the two surgery groups was analyzed by Mann-Whitney test for continuous variable and ordinal variables, by Chi-square test or Fisher exact test for binomial variables

### Detection of CTCs

A total of 220 peripheral blood samples obtained from 110 patients were analyzed. Among the 110 samples collected before surgery, there were 26 (23.64%) which had detectable CTCs and the CTC number ranged from 0.91 to 26.49 (median: 1.76). For the endoscopic group and the open group, there were 13 (24.53%) and 13 (22.81%) samples which had detectable CTC, respectively. The median CTC number was 0.27 (range: 0 to 26.49) in the endoscopic group and 0.21 (range: 0 to 15.16) in the open group. After surgery, 34 (30.91%) out of the 110 patients had detectable CTCs with a median of CTC number as 1.75 (range: 0.98 to 10.56). Of these 34 patients, 15 (15/53, 28.30%) patients underwent endoscopic surgery and 19 (19/57, 33.33%) underwent open surgery. The median CTC number was 0.36 (range: 0 to 10.56) after surgery in the endoscopic group and 0.43 (range: 0 to 9.00) in the open group, respectively. As can be seen in Tables 2 and 3, there was a suggestive increased trend for the CTCs positive rate and the CTC number after operation, although the difference was not statistically significant.

**Table 2:** Comparison for the positive rate of CTCs before and after surgery in the endoscopic and the open group

Study period	Endoscopic group (n = 53)	Open group (n = 57)	*P v*alue ^a^
Before surgery	13(24.53)	13(22.81)	0.832
After surgery	15(28.30)	19(33.33)	0.568
*P v*alue ^b^	0.804	0.327	

^a^Comparison between endoscopic group and open group by Chi-square test

^b^Comparison between preoperation and postoperation period by McNemar test

**Table 3:** Comparison for number of CTCs before and after surgery in the endoscopic and the open group

Study period	Endoscopic group (*n* = 53)	Open group (*n* = 57)	*P v*alue ^a^
Before surgery	0.27(0.004–0.93)	0.21(0.0001–0.75)	0.361
After surgery	0.36(0.002–1.10)	0.43(0.02–1.43)	0.515
*P v*alue ^b^	0.717	0.122	

The number of CTCs was represented as “median (25th and 75th percentiles)”

^a^Comparison between endoscopic group and open group by Mann-Whitney test

^b^Comparison between preoperation and postoperation period by Wilcoxon signed rank test

### Micrometastatic risk of CTCs in different breast cancer surgeries

Using our detecting limit as a cut-off, surgery is considered to implement an increased micrometastatic risk if the number of CTCs is increased beyond this cut-off after operation. In the open surgical group, 17 out of 57 patients (29.82%) showed an increased risk, compared to 8 out of 53 patients (15.09%) in the endoscopic group (χ^2^ = 3.393, *P* = 0.065). Stratified analysis by tumor size, lymph node status and tumor stage also showed that the risk in the open surgical group is higher (OR = 2.5, 2.2 and 2.3, respectively; see Additional file 1: Table S2). Furthermore, propensity score matching was done and 32 pairs of patients were selected. Within the 32 pairs, the open surgical group had a higher proportion of metastasis (OR = 8, *P*_Mc Nemar_ = 0.039; see Additional file 1: Table S3). After adjustment for the potential confounders, the conventional open surgery was associated with a significantly higher micrometastatic risk compared to the endoscopic approach (OR = 2.60, 95%CI: 0.99–6.80; *P* = 0.05; see Table 4). Among the adjusted co-variates, the menopausal status and age also remained in the binary logistic regression model of backward likelihood method. Postmenopausal was associated with higher micrometastatic risk while elder age was associated with lower risk. When the interaction of surgery type and pre-operation CTC status was further considered, the higher risk for the open surgery remained (OR = 3.19, 95%CI: 1.05–9.65; *P* = 0.04), but the interaction was not statistically significant (*P* = 0.191). The uni-variate analysis result and the full-model result were also respectively represented in Table 4 and Additional file 1: Table S4, and the results were similar with the results of the backward-method results.

**Table 4:** The association between surgery type and micrometastatic risk: multi-variate analysis

Variable	Model 0 ^a^		Model 1 ^b^		Model 2 ^c^	
	OR (95% CI)	*P* value	OR (95% CI)	*P* value	OR (95% CI)	*P* value
Surgery type	2.39 (0.93, 6.13)	0.070	2.60 (0.99, 6.80)	0.052	3.19 (1.05, 9.65)	0.040
Menopausal status	0.64 (0.25, 1.65)	0.355	2.16 (0.79, 5.87)	0.131	0.15 (0.02, 1.12)	0.064
Age	1.07 (0.68, 1.67)	0.769	0.18 (0.03, 1.21)	0.077	2.23 (0.79, 6.28)	0.130
Pre-surgery CTC status	0.37 (0.10, 1.35)	0.131	–	–	6.31 (0.10, 418.40)	0.389
Tumor size	1.15 (0.46, 2.87)	0.761	–	–	–	–
Lymph node	1.42 (0.86, 2.33)	0.173	–	–	–	–
Tumor stage	1.41 (0.78, 2.55)	0.261	–	–	–	–
Histology type	- ^e^	0.999	–	–	–	
ER	1.84 (0.72, 4.73)	0.205	–	–	–	–
PR	1.33 (0.54, 3.28)	0.529	–	–	–	–
HER-2	0.91 (0.61, 1.38)	0.669	–	–	–	–
Interaction ^d^	–	–	–	–	0.15 (0.01, 2.56)	0.191
Final model	–	–	–	0.057	–	0.025

OR indicates odds ratio

^a^Uni-variate logistic analysis

^b^Analyzed variables included: surgery type, menopausal status, age, tumor size, lymph node, tumor stage, histology type, estrogen receptors status, progesterone receptors status and HER-2 status. Analyzed by binary logistic regression (backward likelihood method). Only the variables remained in the final model were listed in the table

^c^Additionally included pre-surgery CTC status and its interaction with surgery type based on model 1. Surgery type, pre-surgery CTC status and their interaction were enforcedly remained in the model

^d^The interaction of surgery type and pre-surgery CTC status

^e^The range was too large to be appropriately represented

## Discussion

Our study shows that a suggestive increased trend for the CTC positive rate (23.64% V.S. 30.91%), either in the open surgery group (22.81% V.S. 33.33%) or in the endoscopic surgery group (24.53% V.S. 28.30%). There is also a suggestive increase in the number of CTCs in each group after surgery (open surgery: 0.21 V.S. 0.43; endoscopic surgery: 0.27 V.S. 0.36). Furthermore, higher micrometastatic risk related to CTC was found in the open surgery group compared to the endoscopical group (OR = 3.19, 95%CI: 1.05–9.65), which indicates that patients who underwent open surgery will take more surgery-related potential metastatic risk.

**Fig. 1:**
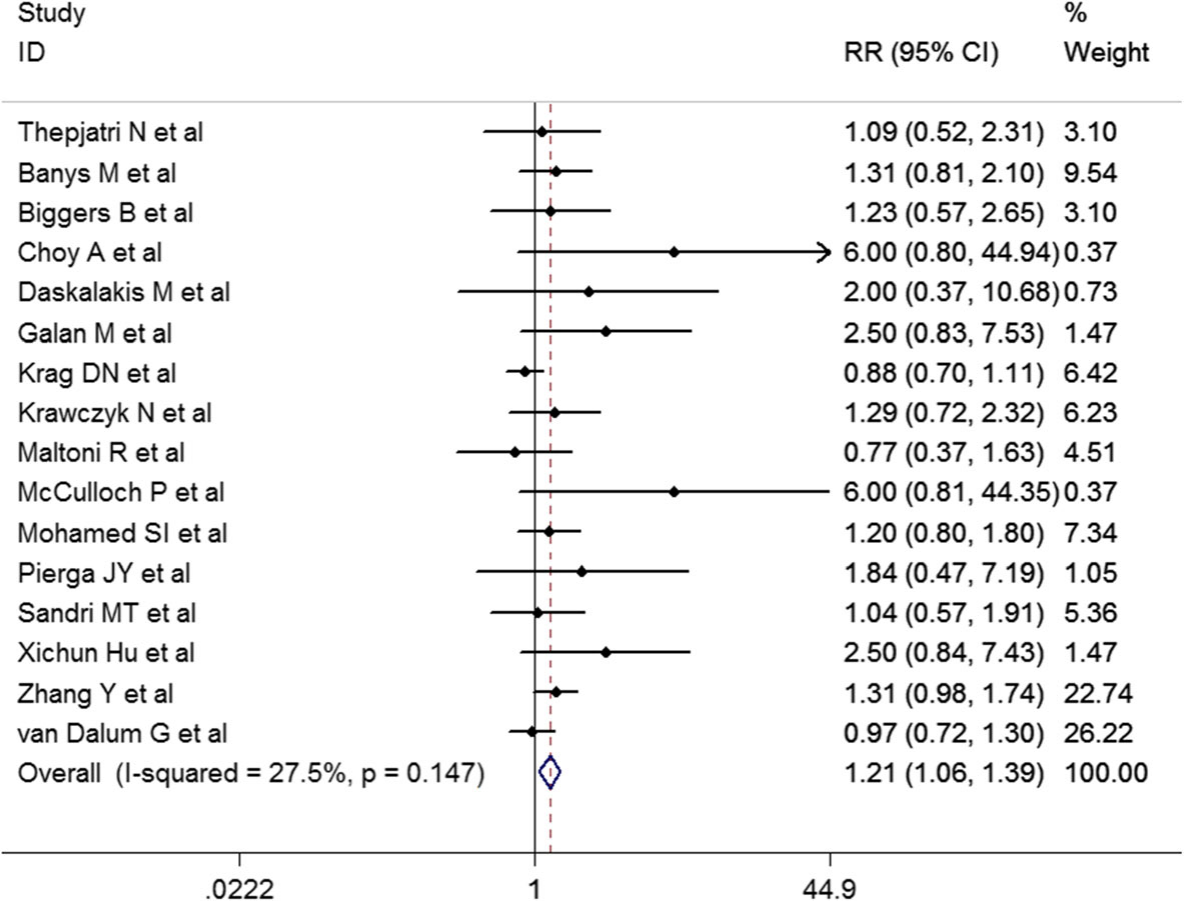
Comparison of CTC positive rate before and after breast cancer surgery: meta-analysis. The heterogeneity of the recruited studies was estimated by the Cochran’s Q test (*P* = 0.147) and the I2 (27.5%). *P* value < 0.10 or I2 over 50% was defined as substantial heterogeneity. Hence, no substantial heterogeneity was observed among the studies and fixed model was used to analyze the overall difference of CTC positive rate before and after breast cancer surgery therapy (OR = 1.21, 95% CI: 1.06–1.39; *P* = 0.006). OR over 1 indicates increased CTC positive rate after surgery compared to preoperation

Previous clinical investigations have suggested that CTC is a prognostic indicator in primary and metastatic breast cancer patients [3, 4]. Monitoring the emergence and dynamics of CTC may provide a more precise assessment for a further personalized cancer treatment. It has long been thought that manipulation of malignant tumors encourages tumor cell dissemination. Animal studies has shown that malignant cells are shed into the blood stream during surgical manipulation of a primary tumor, even leading to an increased incidence of distant metastases [13]. A higher detection rate of CTC has been demonstrated in the blood of patients with colorectal, prostatic cancers or other malignant tumors after surgical procedures [9–12]. However, it seems that there are less studies on the CTC status throughout surgery of breast cancer. In a previous meta-analysis of 194 breast cancer cases, our research group reported an suggestive but not significant increase of CTC-positive rate after surgery [5]. Furthermore, here we reviewed a total of 16 studies with 1553 cases published since 1990 comparing incidence of CTC before and after breast cancer surgery (Table 5, Fig. 1), and found that post-operation CTC-positive rate was higher than the pre-operation condition (OR = 1.21, 95% CI: 1.06–1.39; *P* = 0.006). All these results were in coincidence with the present study suggesting that surgery for breast cancer may induce increase of CTC. There is substantial variation of preoperative and postoperative CTC positivity rate separately among the different publications recruited in our meta-analysis. This may be attributed to the difference of CTC-detection techniques and case-recruitment criteria adopted by different research groups. Especially, since a golden standard of CTC detection method is not available by now, this may introduce a systemic error to the attempt for integrating the numerous publications, and consequently lead to an underestimation of the association between the surgical type and the micrometastatic risk in BC patients in the present study. Unification of the CTC detection method in the future would help to quantitatively and precisely estimate the effect of surgery on the micrometastatic risk.

**Table 5:** Review of 16 published studies of CTC before and after surgery therapy

Author	Year	N	Detection rate preoperatively	Time interval after surgery	Detection rate postoperatively
Zhang Y et al. [2]	2017	286	62/286(22%)	3d	81/286(28%)
Maltoni R et al. [18]	2015	48	13/48(27%)	Immediately	9/43(21%)
Pierga JY et al. [19]	2015	42	3/42(7%)	3-4w	5/38(13%)
van Dalum G et al. [4]	2015	403	75/403(19%)	1w	66/367(18%)
Banys M et al. [20]	2012	209	26/209(12%)	2-3d	34/209(16%)
Daskalakis M et al. [7]	2011	104	2/104(2%)	Immediately	4/104(4%)
Sandri MT et al. [21]	2010	56	16/56(29%)	5d	14/47(30%)
Biggers B et al. [22]	2009	41	10/41(24%)	14d	9/30(30%)
Thepjatri N et al. [23]	2008	30	10/30(33%)	14d	8/22(36%)
Krawczyk N et al. [24]	2008	130	17/130(13%)	Immediately	22/130(17%)
Ismail MS et al. [25]	2004	41	20/41(49%)	Immediately	24/41(59%)
Hu XC et al. [6]	2003	49	4/49(8%)	1d	10/49(20%)
Galan M et al. [26]	2002	59	4/59(7%)	1d	10/59(17%)
Krag DN et al. [8]	1999	21	18/19(95%)	2 h	15/18(83%)
Choy A et al. [27]	1996	18	1/18(5%)	Immediately	6/18(33%)
McCulloch P et al. [28]	1995	16	1/16(6%)	Immediately	6/16(38%)

The operative treatment of breast cancer has made substantial directional changes. From Halsted’s radical operation to classical modified radical mastectomy, to widely accepted breast conservation, a shift from “maximum tolerable” to “minimum effective” treatment strategy has been evident [29, 30]. Conventional mastectomy is often associated with complications such as an obvious scar, physical defect, upper extremity edema and dysfunction. Mastectomy patients tend to suffer from ongoing anxiety and depression over the disfigurement of their bodies and the loss of a feminine figure. The endoscopic approach emerges as a response to the need for new “minimally invasive” surgical techniques that assure a local tumor control while decreasing surgery-related morbidity [31, 32]. Under endoscopic vision, meticulous dissection and hemostasis can be achieved with the harmonic scalpel, and the average blood loss is less than the conventional method [33]. Endoscopic breast surgery plays an important role in the treatment of patients with early breast cancer. It is suitable for patients with small-to-moderate-sized breasts and reconstruction can be performed immediately or at a second stage, especially for Asian women. Endoscopic nipple and/or skin sparing subcutaneous mastectomy with immediate breast reconstruction restore the patient’s body image and improve cosmesis over open surgery. For instance, in our institution, we performed endoscopic harvesting of latissimus dorsi flap with prosthesis implantation for immediate breast reconstruction after nipple-sparing mastectomy in 2 patients with breast cancer who wished to avoid a long scar on the back. Both patients were satisfied with the esthetic results of the procedure using only 3 small trocar ports instead of a long visible scar on the back [34].

However, evidence for the safety in support of this technique is lacing. Whether the minimization of invasion by endoscopic surgery leads to a less or more induction of CTCs release is unknown by now. To the best of our knowledge, the present study is the first clinical study evaluating the effect of endoscopic surgery on CTCs in breast cancer patients underwent surgery. When increased micrometastatic risk is defined as the CTC number increased beyond the detection cut-off after operation, less micrometastatic risk was found in the endoscopic operated group, which indicates that patients underwent open surgery will take more surgery-related potential metastatic risk. Although the surgical principles are the same for both surgical approaches, the surgical sequence is different from each other. This difference may partially block cancer cell dissemination enhanced by surgical manipulation. However, a study with long-term follow-up in a larger population is required to confirm the clinical indication of our results. Our study found no significant difference of surgery duration or bleeding volume between the micrometastatic patients and the non-micrometastatic patients. It may indicate that these characteristics are not the main contributor for the higher micrometastatic risk in the open surgery group. The present study has several major limitations. First, the study is of observational design. Bias introduced by potential confounder, such as the subjective decision of therapy strategy affected by the disease status, could not be completed ruled out. Randomized clinical trial would be needed in the future to validate our findings. Second, some specific types of breast cancer such as ductal carcinoma was not recruited with adequate sample size for subgroup analysis. Hence the increase of micrometastatic risk observed in the present study may not be generalized to all cases of breast cancer. Third, although the increase of CTC in peripheral blood was widely accepted to be a promising biomarker of poorer prognosis, the clinical outcome of the patients, such as disease free survival and metastasis rate was not investigated in the present study due to the limited follow-up period and sample size. Whether the long-term risk of endoscopic breast cancer surgery is comparable with open surgery for breast cancer still acquires further study.

## Conclusions

The present study found a suggestive increase of CTCs in breast cancer patients after surgery, and the endoscopic surgery was associated with less risk of metastasis related to CTCs compared to open radical mastectomy. The results may indicate that monitoring of CTC status is necessary for the breast cancer patients after surgery, especially for those who underwent open surgery. Future studies are needed to confirm our finding.

## Supplementary information

**Additional file 1: Table S1.** Comparison of surgery duration and bleeding volume between the micrometastatic patients and the non-micrometastatic patients. **Table S2.** Stratified analysis of surgery-specific micrometastatic risk by tumor size, lymph node and tumor stage. **Table S3.** Propensity score matching analysis of micrometastasis between different surgery groups. **Table S4.** The association between surgery type and micrometastatic risk: full model of multi-variate analysis.

## Data Availability

The datasets used and/or analysed during the current study are available from the corresponding author on reasonable request.

## Abbreviations

CK-19: cytokeratin-19
CTC: circulating tumor cell
MCF-7: Michigan Cancer Foundation-7 breast cancer cell line

## References

[1] Bray F, Ferlay J, Soerjomataram I, Siegel RL, Torre LA, Jemal A (2018). Global cancer statistics 2018: GLOBOCAN estimates of incidence and mortality worldwide for 36 cancers in 185 countries. CA Cancer J Clin.

[2] Zhang Y, Lv Y, Niu Y, Su H, Feng A (2017). Role of circulating tumor cell (CTC) monitoring in evaluating prognosis of triple-negative breast Cancer patients in China. Med Sci Monit.

[3] Magbanua MJ, Carey LA, DeLuca A, Hwang J, Scott JH, Rimawi MF (2015). Translational breast Cancer research consortium. Circulating tumor cell analysis in metastatic triple-negative breast cancers. Clin Cancer Res.

[4] van Dalum G, van der Stam GJ, Tibbe AG, Franken B, Mastboom WJ, Vermes I (2014). Circulating tumor cells before and during follow-up after breast cancer surgery. Int J Oncol.

[5] Yan WT, Cui X, Chen Q, Li YF, Cui YH, Wang Y (2017). Circulating tumor cell status monitors the treatment responses in breast cancer patients: a meta-analysis. Sci Rep.

[6] Hu XC, Loo WT, Chow LW (2003). Surgery-related shedding of breast cancer cells as determined by RT-PCR assay. J Surg Oncol.

[7] Daskalakis M, Mavroudis D, Sanidas E, Apostolaki S, Askoxylakis I, de Bree E (2011). Assessment of the effect of surgery on the kinetics of circulating tumour cells in patients with operable breast cancer based on cytokeratin-19 mRNA detection. Eur J Surg Oncol.

[8] Krag DN, Ashikaga T, Moss TJ, Kusminsky RE, Feldman S, Carpet NZ (1999). Breast Cancer cells in the blood: a pilot study. Breast J.

[9] Yu JJ, Xiao W, Dong SL, Liang HF, Zhang ZW, Zhang BX (2018). Effect of surgical liver resection on circulating tumor cells in patients with hepatocellular carcinoma. BMC cancer.

[10] Weitz J, Kienle P, Lacroix J, Willeke F, Benner A, Lehnert T (1998). Dissemination of tumor cells in patients undergoing surgery for colorectal cancer. Clin Cancer Res.

[11] Wei XQ, Ma Y, Chen Y, Liu X, Zhao M, Zhou LW (2018). Laparoscopic surgery for early cervical squamous cell carcinoma and its effect on the micrometastasis of cancer cells. Medicine.

[12] Eschwège P, Dumas F, Blanchet P, Le Maire V, Benoit G, Jardin A (1995). Haematogenous dissemination of prostatic epithelial cells during radical prostatectomy. Lancet.

[13] Nishizaki T, Matsumata T, Kanematsu T, Yasunaga C, Sugimachi K (1990). Surgical manipulation of VX2 carcinoma in the rabbit liver evokes enhancement of metastasis. J Surg Res.

[14] Leff DR, Vashisht R, Yongue G, Keshtgar M, Yang GZ, Darzi A (2011). Endoscopic breast surgery: where are we now and what might the future hold for video-assisted breast surgery?. Breast Cancer Res Treat.

[15] Reinholz MM, Kitzmann KA, Tenner K, Hillman D, Dueck AC, Hobday TJ, Reinholz MM, Kitzmann KA, Tenner K (2011). Cytokeratin-19 and mammaglobin gene expression in circulating tumor cells from metastatic breast cancer patients enrolled in north central Cancer treatment group trials, N0234/336/436/437. J Surg Res.

[16] Wang L, Wang Y, Liu Y, Cheng M, Wu X, Wei H (2009). Flow cytometric analysis of CK19 expression in the peripheral blood of breast carcinoma patients: relevance for circulating tumor cell detection. J Exp Clin Cancer Res.

[17] Stathopoulou A, Gizi A, Perraki M, Apostolaki S, Malamos N, Mavroudis D (2003). Real-time quantification of CK-19 mRNA-positive cells in peripheral blood of breast cancer patients using the lightcycler system. Clin Cancer Res.

[18] Maltoni R, Fici P, Amadori D, Gallerani G, Cocchi C, Zoli M (2015). Circulating tumor cells in early breast cancer: a connection with vascular invasion. Cancer Lett.

[19] Pierga JY, Petit T, Lévy C, Ferrero JM, Campone M, Gligorov J (2015). Pathological response and circulating tumor cell count identifies treated HER2+ inflammatory breast cancer patients with excellent prognosis: BEVERLY-2 survival data. Clin Cancer Res.

[20] Banys M, Krawczyk N, Becker S, Jakubowska J, Staebler A, Wallwiener D (2012). The influence of removal of primary tumor on incidence and phenotype of circulating tumor cells in primary breast cancer. Breast Cancer Res Treat.

[21] Sandri MT, Zorzino L, Cassatella MC, Bassi F, Luini A, Casadio C (2010). Changes in circulating tumor cell detection in patients with localized breast cancer before and after surgery. Ann Surg Oncol.

[22] Biggers B, Knox S, Grant M, Kuhn J, Nemunatitis J, Fisher T (2009). Circulating tumor cells in patients undergoing surgery for primary breast cancer: preliminary results of a pilot study. Ann Surg Oncol.

[23] Thepjatri N, Kuhn JA, Knox SM, Grant MD, Nemunaitis JJ, Fisher TL (2008). Preliminary results of a pilot study of circulating tumor cells in patients undergoing surgery for primary breast cancer. Ann Surg Oncol.

[24] Krawczyk N, Fehm T, Banys M, Duerr-Stoerzer S, Wallwiener D, Solomayer EF. Comparison between circulating and disseminated tumor cells in breast cancer. Eur J Cancer 2008; Supplements 6(7): 140.

[25] Ismail MS, Wynendaele W, Aerts JL, Paridaens R, Gaafar R, Shakankiry N (2004). Detection of micrometastatic disease and monitoring of perioperative tumor cell dissemination in primary operable breast cancer patients using real-time quantitative reverse transcription-PCR. Clin Cancer Res.

[26] Galán M, Viñolas N, Colomer D, Soler G, Muñoz M, Longarón R (2002). Detection of occult breast cancer cells by amplification of CK19 mRNA by reverse transcriptase-polymerase chain reaction: role of surgical manipulation. Anticancer Res.

[27] Choy A, McCulloch P (1996). Induction of tumour cell shedding into effluent venous blood breast cancer surgery. Br J Cancer.

[28] McCulloch P, Choy A, Martin L (1995). Association between tumour angiogenesis and tumour cell shedding into effluent venous blood during breast cancer surgery. Lancet.

[29] Plesca M, Bordea C, El Houcheimi B (2016). Evolution of radical mastectomy for breast cancer. J Med Life.

[30] Sakorafas GH, Safioleas M (2010). Breast cancer surgery: an historical narrative. Part III. From the sunset of the 19th to the dawn of the 21st century. Eur J Cancer Care.

[31] Takahashi H, Fujii T, Nakagawa S, Inoue Y, Akashi M, Toh U (2014). Usefulness of endoscopic breast-conserving surgery for breast cancer. Surg Today.

[32] Lai HW, Chen ST, Chen DR, Chen SL, Chang TW, Kuo SJ (2016). Current trends in and indications for endoscopy-assisted breast surgery for breast Cancer: results from a six-year study conducted by the Taiwan endoscopic breast surgery cooperative group. PLoS One.

[33] Jiang J, Yang XH, Fan LJ, Zhang Y, Zhang F, Zhou Y (2005). Application of endoscopy-assistant operation in surgical treatment of breast diseases. Zhonghua Yi Xue Za Zhi.

[34] Xu S, Tang P, Chen X, Yang X, Pan Q, Gui Y (2016). Novel technique for laparoscopic harvesting of latissimus dorsi flap with prosthesis implantation for breast reconstruction: a preliminary study with 2 case reports. Medicine (Baltimore).

