# Supplementary material for: Less micrometastatic risk related to circulating tumor cells after endoscopic breast cancer surgery compared to open surgery
Source: BMC Cancer. 2019 Nov 8;19:1070. doi: 10.1186/s12885-019-6158-3 (PMC6842272; doi:10.1186/s12885-019-6158-3)
Supplement: Supplementary file 1 — Additional file 1: Table S1. Comparison of surgery duration and bleeding volume between the micrometastatic patients and the non-micrometastatic patients. Table S2. Stratified analysis of surgery-specific micrometastatic risk by tumor size, lymph node and tumor stage. Table S3. Propensity score matching analysis of micrometastasis between different surgery groups. Table S4. The association between surgery type and micrometastatic risk: full model of multi-variate analysis. [file 12885_2019_6158_MOESM1_ESM.docx]

**Additional file 1: Table S1.** Comparison of surgery duration and bleeding volume between the micrometastatic patients and the non-micrometastatic patients.

| Characteristic | Micrometastatic  (n=25) | Non-micrometastatic  (n=85) | *P* value ^a^ |
| --- | --- | --- | --- |
| Surgery duration, min | 140(66-303) | 174(75-479) | 0.096 |
| Bleeding volume, mL | 100(30-250) | 100(30-800) | 0.470 |

^a^ Analyzed by Mann-Whitney test.

**Table S2.** Stratified analysis of surgery-specific micrometastatic risk by tumor size, lymph node and tumor stage.

| Characteristic | Stratum | Endoscopic group  (n=53) | Open group  (n=57) | Odds ratio ^a^ | *P* value ^a^ |
| --- | --- | --- | --- | --- | --- |
| **Tumor size** |  |  |  | 2.5(0.9, 6.4) | 0.067 |
|  | **T_1_** |  |  |  |  |
|  | No microstasis | 36(81.8%) | 29(72.5%) |  |  |
|  | Microstasis | 8(18.2%) | 11(27.5%) |  |  |
|  | **T_2-3_** |  |  |  |  |
|  | No microstasis | 9(100%) | 11(64.7%) |  |  |
|  | Microstasis | 0(0%) | 6(35.3%) |  |  |
| **Lymph node(pN)** |  |  |  | 2.2(0.8, 5.7) | 0.107 |
|  | **pN_0_** |  |  |  |  |
|  | No microstasis | 30(90.9%) | 21(75.0%) |  |  |
|  | Microstasis | 3(9.1%) | 7(25.0%) |  |  |
|  | **pN_1-3_** |  |  |  |  |
|  | No microstasis | 15(75.0%) | 19(65.5%) |  |  |
|  | Microstasis | 5(25.0%) | 10(34.5%) |  |  |
| **Tumor stage** |  |  |  | 2.3(0.9, 5.9) | 0.089 |
|  | **Ⅰ** |  |  |  |  |
|  | No microstasis | 26(89.7%) | 20(76.9%) |  |  |
|  | Microstasis | 3(10.3%) | 6(23.1%) |  |  |
|  | **Ⅱ-Ⅲ** |  |  |  |  |
|  | No microstasis | 19(79.2%) | 20(64.5%) |  |  |
|  | Microstasis | 5(20.8%) | 11(35.5%) |  |  |

^a^ Analyzed by Mantel-Haenszel method.

**Table S3.** Propensity score matching analysis of micrometastasis between different surgery groups.

|  |  | Open surgery group | |
| --- | --- | --- | --- |
|  |  | Micrometastatic | Non-micrometastatic |
| Microscopic group | Micrometastatic | 3 | 1 |
|  | Non-micrometastatic | 8 | 20 |

Odds ratio=8; PMc Nemar=0.039.

**Table S4.** The association between surgery type and micrometastatic risk: full model of multi-variate analysis.

| Variable | Model 1 | | |  | | | Model 2 | | | | |  |
| --- | --- | --- | --- | --- | --- | --- | --- | --- | --- | --- | --- | --- |
|  | OR (95% CI) | *P* value | |  | | | OR (95% CI) | | | *P* value | |  |
| Surgery type | 2.54 (0.89, 7.21) | | 0.081 | |  | | | 4.08 (1.19, 13.98) | | | 0.025 | |
| Menopausal status | 0.14 (0.02, 1.00) | | 0.050 | |  | | | 0.13 (0.02, 0.99) | | | 0.049 | |
| Age | 2.26 (0.82, 6.20) | | 0.114 | | |  | | | 2.21 (0.79, 6.19) | | 0.132 | |
| Pre-surgery CTC status | - | | - | |  | | | 17.5 (0.18, 1671.4) | | | 0.219 | |
| Tumor size | 0.93 (0.27, 3.16) | | 0.904 | |  | | | 0.55 (0.14, 2.11) | | | 0.385 | |
| Lymph node | 1.69 (0.35, 8.22) | | 0.513 | |  | | | 1.26 (0.25, 6.34) | | | 0.781 | |
| Tumor stage | 0.78 (0.11, 5.71) | | 0.810 | |  | | | 1.34 (0.16, 10.85) | | | 0.786 | |
| Histology type | - ^b^ | | 0.999 | |  | | | - ^e^ | | | 0.999 | |
| ER | 2.41 (0.66, 8.76) | | 0.182 | |  | | | 2.22 (0.61, 8.08) | | | 0.227 | |
| PR | 0.91 (0.27, 3.06) | | 0.883 | |  | | | 0.89 (0.26, 3.02) | | | 0.857 | |
| HER-2 | 1.05 (0.67, 1.66) | | 0.826 | |  | | | 1.01 (0.64, 1.61) | | | 0.953 | |
| Interaction ^a^ | - | | - | |  | | | 0.08 (0.004, 1.85) | | | 0.115 | |
| Final model | - | | 0.163 | |  | | | - | | | 0.096 | |

OR indicates odds ratio.

^a^ The interaction of surgery type and pre-surgery CTC status.

^b^ The range was too large to be appropriately represented.
